# Total Endoscopic or Endoscope-Assisted Excision of Non-embolized Advanced Juvenile Nasopharyngeal Angiofibroma: A Clinical Case Series

**DOI:** 10.7759/cureus.60747

**Published:** 2024-05-21

**Authors:** Nazneen Liaqat, Israr Ud Din, Ihtisham Ul Haq, Shakir Ullah, Izhar Ahmad, Imran Khan

**Affiliations:** 1 Department of Otorhinolaryngology-Head and Neck Surgery, Khyber Teaching Hospital (KTH) Medical Teaching Institute, Peshawar, PAK

**Keywords:** radkowski, endoscope-assisted, endoscope, non-embolized, juvenile nasopharyngeal angiofibroma

## Abstract

Background

Surgical excision is the primary treatment for juvenile nasopharyngeal angiofibroma (JNA), but this procedure is challenging due to its high vascularity and local aggressiveness. Moreover, preoperative embolization is a subject of debate.

Objective

The objective of this study is to assess the efficacy, safety, and feasibility of endoscope-assisted excision as a surgical intervention for non-embolized advanced JNA.

Materials and methods

This case series involved six male patients (mean age: 16 years) with JNA, classified as stages Ⅱc to Ⅲb according to the Radkowski classification. None underwent preoperative embolization.

Results

Two stage Ⅱc cases underwent total endoscopic endonasal excision. One patient with stage Ⅲa and another with stage Ⅲb underwent surgery via an endoscope-assisted sublabial approach. Two patients, one with stage Ⅱc JNA and another with Ⅲb, underwent a two-stage procedure. Postoperative CT scans showed no residual disease at the six-month mark. On average, each procedure required 1.5 units of blood transfusion. One patient experienced intraoperative bleeding, whereas the remaining patients were free of any major complications. The mean operation duration was 175 minutes per procedure. The mean length of stay at the hospital was 3.75 days per procedure.

Conclusion

Endoscope-assisted or purely endoscopic approaches can be safely and effectively employed for the complete excision of non-embolized advanced JNAs.

## Introduction

Juvenile nasopharyngeal angiofibroma (JNA), with an incidence of 1:150,000, is a benign yet highly vascular tumor associated with significant mortality and morbidity [1]. Surgical excision, the gold standard treatment for JNA, is highly challenging due to its high vascularity and local aggressiveness [2]. These tumors have an intricate vascular supply, deriving blood from both the external and internal carotid arteries [3]. Furthermore, JNAs exhibit aggressive invasion into neighboring structures, complicating surgical resection. Despite technological advances and improved perioperative care, the complex skull anatomy and the vascularity of JNAs contribute to the continued difficulty of these operations [2]. Preoperative embolization is generally considered to be effective at reducing blood loss and the need for blood transfusions during and after surgery [4]. Moreover, it also reduces the operative time [5] and length of hospital stay [4]. However, its safety and absolute need remain the subjects of debate and controversy. The distortion of tumor boundaries during embolization may contribute to incomplete resection and a potential increase in tumor recurrence rates [1]. Furthermore, embolization poses a risk of embolic complications, potentially causing cranial nerve lesions, subdural hematoma [1], and vision loss [6], particularly in patients with anastomoses between the external carotid and internal carotid arteries [5]. A higher cost and inconsistent availability are some of the additional challenges faced by this procedure [7]. The major obstacle faced by authors for preoperative embolization, in this case series, was its non-availability and higher cost of referral. This case series aimed to conduct a comprehensive assessment of the efficacy, safety, and feasibility of endoscope-assisted excision as a surgical intervention for non-embolized JNAs, stages Ⅱc to Ⅲb, as classified by the Radkowski classification system.

## Materials and methods

This study was conducted at Khyber Teaching Hospital, Peshawar, Pakistan. The ethical approval was obtained from the Institutional Research and Ethical Review Board of Khyber Medical College, Peshawar, Pakistan (approval number: 762/DME/KMC). From the pool of surgically treated JNA patients, from January 2023 to July 2023, those cases who were classified by contrast-enhanced CT/MRI scan as stages Ⅱc to Ⅲb according to the Radkowski classification and who had undergone endoscopic or endoscope-assisted excisions were selected. Information, including preoperative investigations, imaging, staging, the volume of blood transfused, the length of stay at the hospital, the duration of the procedure, and complications, if any, was obtained. All the patients have been kept under close monthly follow-up. Detailed nasal examinations and nasoendoscopy are performed during these visits. Additionally, postoperative contrast-enhanced CT scans were taken in the second and then sixth months to assess the presence of residual disease.

Surgical technique

In all the patients, under direct visualization with a zero-degree endoscope, 2% lignocaine with 1:200,000 epinephrine was injected into the nasal floor, ipsilateral gingivolabial groove, lateral nasal wall, and nasal septum (wherever needed). The nasal cavity was packed with Merocel soaked in epinephrine. Reverse Trendelenburg position with hypotensive anesthesia was maintained throughout the procedure. A septal correction was performed in patients with nasal septal deviation. Posterior septectomy was conducted, wherever needed, and it was reconstructed at the end of the procedure.

With an endoscopic-endonasal approach, inferior turbinectomy was performed. Intranasal incisions were made in the lateral nasal wall, to obtain a modified Denker’s approach. In patients with narrow endonasal exposure, an additional sublabial approach was taken to expose the anterior maxillary wall. The lateral nasal wall was exposed through subperiosteal dissection. A bony window was made in the medial maxillary wall using osteotomes and was extended to the anteromedial aspect of the sinus. A wide medial maxillectomy was performed. The nasolacrimal duct was cut obliquely.

**Figure 1:**
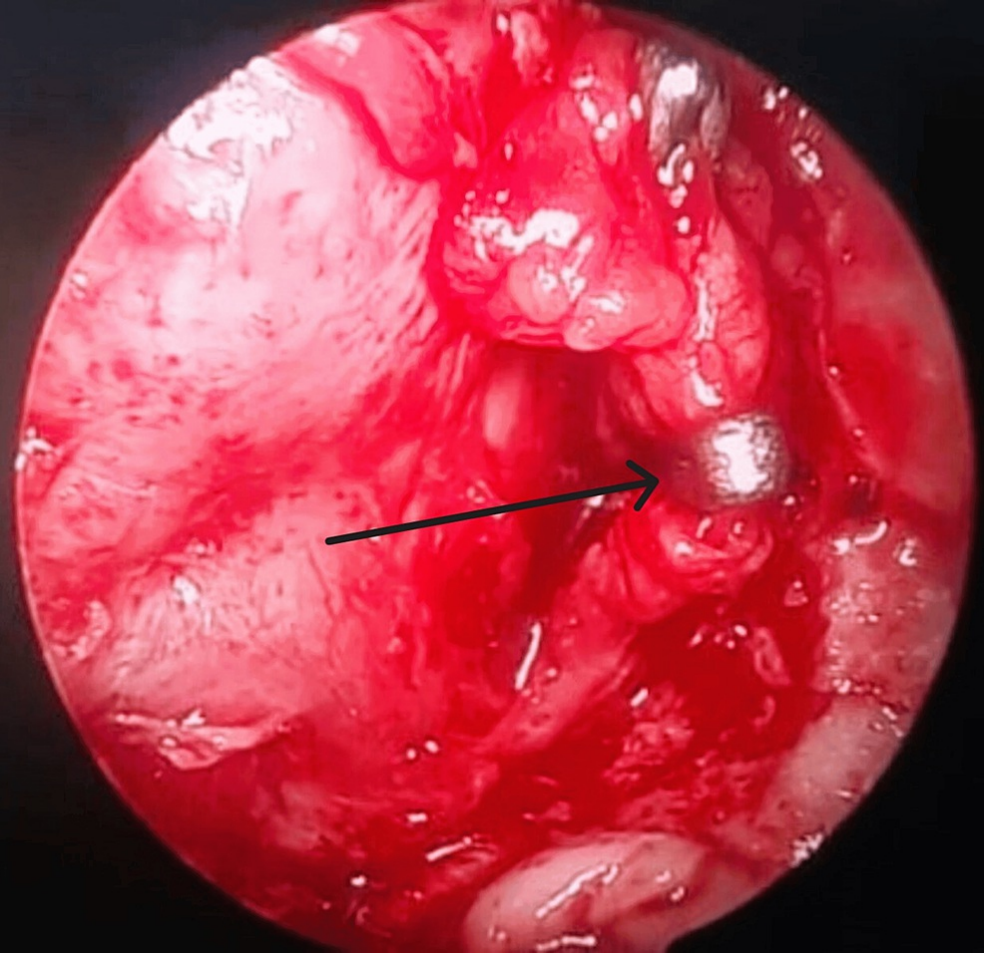
Intraoperative endoscopic view of clipped left internal maxillary artery, through a posterior maxillary wall, lying in the left pterygopalatine fossa

Using an endoscopic-endonasal bi-nostril, four-handed technique, the posterior wall of the maxillary sinus was removed using a mallet and gouge or bone punch in a controlled manner, to expose the pterygopalatine fossa. It was observed that the larger the size and the deeper the extent of disease into the infratemporal fossa, the easier it was to expose the pterygopalatine fossa through a posterior maxillary wall. Careful dissection was performed to expose the internal maxillary artery, which was either clipped or cauterized, as shown in Figure 1.

Depending upon its size and extent, the tumor was dissected out, released from its attachments, and pushed inferomedially. Accordingly, sphenoidotomy was performed. In patients with extension into the cranial base or cavernous sinus, the tumor was dissected under direct visualization. Furthermore, the tumor was consistently pushed medially toward the nasopharynx and gradually removed from the infratemporal fossa. It was released from all attachments, resected in an en bloc fashion, and removed through a transoral route. The pterygoid wedge, basisphenoid, and clivus were drilled. Careful inspection was conducted to identify and remove any remnants or gross digitations. Hemostasis was secured meticulously. Throughout the procedure, adrenaline-soaked Merocel packs were used to clear the surgical field and identify the attachment of the JNA in detail.

## Results

This retrospective, descriptive study included six patients, all of whom were male with a mean age of 16 years. All the patients presented with chief complaints of epistaxis and nasal obstruction. In all the patients, preoperative contrast-enhanced CT/MRI scans were performed, and the tumors were classified according to the Radkowski classification. Three patients were classified as stage Ⅱc, one as stage Ⅲa, and two as stage Ⅲb. Notably, none of the patients underwent preoperative embolization. Table 1 depicts the clinical and procedural characteristics of the patients.

**Table 1:** Clinical and procedural characteristics of the patients Stage: Radkowski stage ^a^Underwent radiotherapy at a different center but did not respond M, male; y, year

Patient number	Age/gender	Stage	Approach	Artery clipped	Duration of procedure (hour)	Units of transfusion	Length of stay (days)	Gross total resection	Residual disease	Follow-up (weeks)	Major complication
1	14 y/m	Ⅱc	Endoscopic endonasal	None	03:00	-	3	Yes	Yes	52	None
			Endoscopic+sublabial	None	02:10	1	3	Yes	No		None
2	20 y/m	Ⅱc	Endoscopic endonasal	None	02:50	-	3	Yes	No	40	None
3	34 y/m	Ⅱc	Endoscopic endonasal	None	02:20	-	3	Yes	No	38	None
4	8 y/m	Ⅲa	Endoscopic+sublabial	Internal maxillary artery	03:45	1	4	Yes	No	47	None
5	11 y/m^a^	Ⅲb	Endoscopic+sublabial	Internal maxillary artery	03:30	2	4	Yes	No	48	None
6	11 y/m	Ⅲb	Endoscopic endonasal	None	03:45	3	6	No	Yes	30	Intraoperative bleed
			Endoscopic+sublabial	Internal maxillary artery	02:00	1	4	Yes	No		None

**Figure 2:**
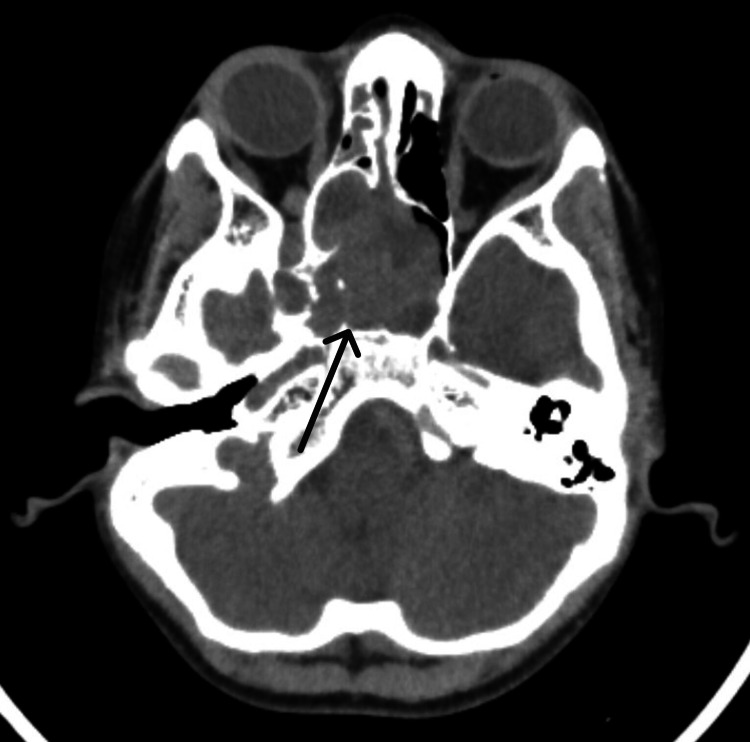
Preoperative CT scan of patient number 6 showing stage Ⅲb juvenile nasopharyngeal angiofibroma

**Figure 3:**
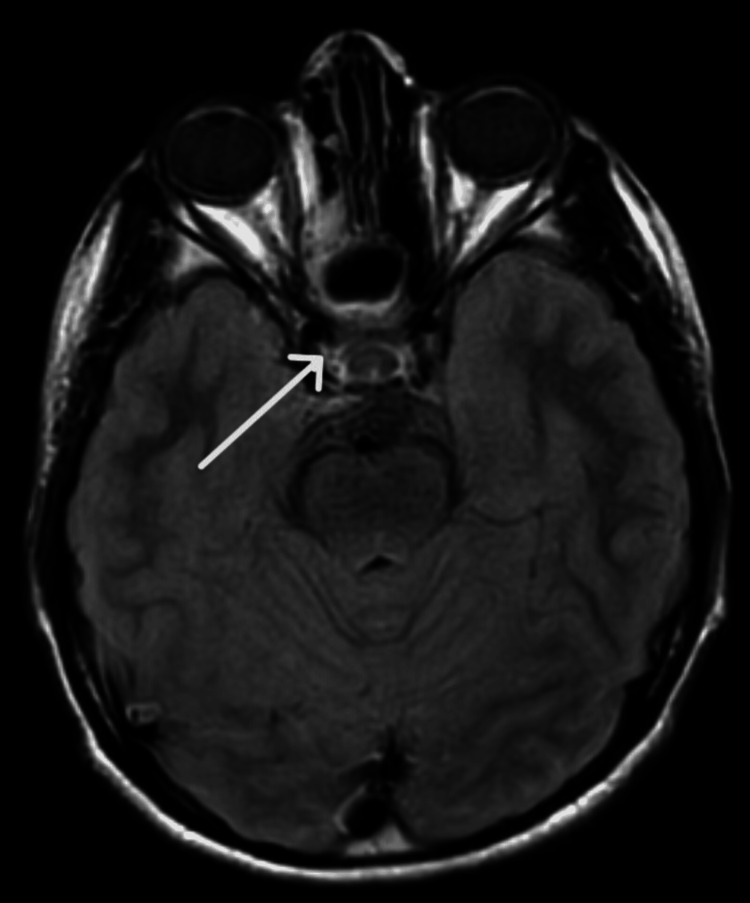
Postoperative second-month contrast-enhanced MRI scan of patient number 6 showing the complete excision of juvenile nasopharyngeal angiofibroma

All patients have been placed under regular follow-up care. Importantly, with a mean follow-up duration of 42.5 weeks, all the patients remained asymptomatic. Postoperative contrast-enhanced CT/MRI scans were performed at the second-month and then at the sixth-month mark. Figure 2 is the preoperative CT scan of patient number 6, showing a hyperdense lesion extending into the right cavernous sinus and thus staged as Ⅲb JNA, while Figure 3 shows the postoperative CT scan of the same patient at the second-month mark, with complete excision.

The required number of units of blood transfusion per procedure ranged from zero to three units. The mean duration of surgical procedures in the present study was 175 minutes. The mean length of stay at the hospital was 3.75 days per procedure. Patient number 6 with stage IIIb JNA experienced intraoperative bleeding as a major surgical complication while removing the mass from the cavernous sinus. The procedure was halted, and it was managed with packing. The excision was completed in the second stage. All the patients tolerated the procedure well.

## Discussion

Surgery has historically been the preferred treatment for JNA. Traditional methods such as transpalatal, lateral rhinotomy, and midfacial degloving have been proven successful; however, they are associated with cosmetic and functional comorbidities [8]. Endoscopic techniques have emerged as the preferred route for the management of JNA due to the lower rate of complications and lesser chances of recurrence [9,10]. Nonetheless, the role of these techniques in treating larger tumors remains controversial. While they may not serve as standalone approaches, there is potential for endoscopic techniques to play a supplementary role alongside open surgical approaches in managing advanced JNA [8,11]. Advanced JNAs with intracranial extension, skull base erosion, and infratemporal extensions are uncommon, and these have significantly greater chances of recurrence as compared to early-stage disease [12,13].

In the present case series, six patients with advanced JNA underwent surgical excision via either endoscopic endonasal or endoscope-assisted sublabial (transmaxillary-infratemporal fossa) approach. None of these patients underwent any preoperative embolization of feeder vessels due to non-availability and the higher cost of referral. Two patients needed a second surgery for the removal of residual disease, while the remaining four had complete excision in a single setting. In patient number 1, the site of residual disease was the pterygoid wedge. While in patient number 6, the JNA extended into the cavernous sinus, which upon removal bled heavily. The procedure was halted, and the excision was completed in the second setting. Regarding patient number 6, radiotherapy was done by an outside hospital as the JNA closely abutted the internal carotid artery system. He presented in the emergency unit with epistaxis. After first aid measures, the multidisciplinary team advised excision, which was proceeded, later on.

Janakiram et al. operated on 15 patients with non-embolized JNA characterized by Radkowski stage Ⅱa and concluded that the endoscopic approach was effective for the excision of small- to medium-sized JNAs, with lesser blood loss and lower rates of recurrence. Intraoperative hemostasis was attained by the ligation of feeder vessels before tumor manipulation [7]. Mohammadi Ardehali et al. performed surgical excision in 32 patients with advanced JNA, spanning Radkowski stages Ⅱc to Ⅲb. Among these, 29 cases were non-embolized. With a mean blood loss of 1,261±893 cc and a recurrence rate of 21.88%, their findings affirmed that endoscope-assisted excision without prior embolization is not only possible but also highly effective in advanced JNA management [8].

The mean duration of surgical procedures in the present study was 175 minutes. Fonseca et al. reported the average time to be 140 minutes [14]. While for Janakiram et al., the procedure took 101 minutes on average [7]. The longer operative time in our study can be attributed to the advanced stage of the disease. According to the current literature, the mean hospitalization time for JNA excision ranges from 3.56 to 3.66 days [7,8]. In the present case series, the mean length of stay was 3.75 days per procedure. Fonseca et al. reported that six out of 15 patients needed intraoperative blood transfusions [14]. However, in the present study, intraoperative transfusion was performed in four out of the six patients.

The loss of sensations due to neurovascular manipulation is one of the major adverse effects of endoscopic approaches [15]. In the present case study, four out of six patients had postoperative temporary numbness in the ipsilateral V2 territory, which was reported to have been improved in the follow-up.

Limitations of study

The authors acknowledge the limitations of this study, which include having a small sample size, being a single-center study, and having a relatively short mean follow-up period of 42 weeks.

## Conclusions

JNAs, though highly vascular and locally invasive, are treatable with complete surgical excision. The endoscopic-endonasal route with the utilization of preoperative embolization provides surgeons with a feasible and effective treatment modality associated with limited morbidity. In situations with advanced JNAs where preoperative embolization is unavailable or contraindicated, the endoscopic approaches can still be employed effectively. Thus, based on the presented case series of six patients, the authors conclude that with pre-surgical multidisciplinary planning, good teamwork, and experience on the part of the surgeon, endoscope-assisted or purely endoscopic approaches can be employed, with both safety and effectiveness, for the complete excision of non-embolized advanced JNAs.
